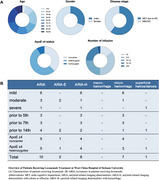# Characteristics of patients on Lecanemab treatment at West China Hospital of Sichuan University: perspectives from real‐world experience.

**DOI:** 10.1002/alz70859_105423

**Published:** 2025-12-26

**Authors:** Ruihan Wang, Qin Chen, Na Hu, Weihong Kuang, Ying Li, Su Lv, Rong Tian, Li Li, Hui Gao, Yingying Tang, Linyuan Qin, Shiyu Feng, Hanlin Cai, Feng Yang, Caimei Luo, Mengyao Guo

**Affiliations:** ^1^ West China Hospital of Sichuan University, Chengdu, Sichuan China

## Abstract

**Background:**

Alzheimer’s disease (AD) poses a significant public health challenge in China. Recent advancements in disease‐modifying therapies, particularly lecanemab, have brought new therapeutic opportunities. In China, nationwide implementation of lecanemab in clinical practice was launched in June 2024. The study aims to investigate the characteristics of patients receiving lecanemab treatment and explore ways to improve patient recruitment and Amyloid‐related imaging abnormalities (ARIA) monitoring.

**Method:**

We retrospectively reviewed patients who received lecanemab treatment at West China Hospital from June 2024 to January 2025 (n=101). Patients interested in lecanemab treatment were screened by a multidisciplinary team (MDT) comprising experts from Neurology, Psychiatry, Geriatrics, Radiology, and Nuclear Medicine. Requirement for referral to the MDT clinic included a diagnosis of mild cognitive impairment (MCI) or mild dementia confirmed by amyloid PET, no evidence of disease other than AD being the primary cause of cognitive impairment, and the ability to undergo MRI scans. ARIA was monitored by comparing MRI scans from the same 3T scanner, interpreted by the same radiologist. In case of suspected adverse reactions, urgent MRI scans were promptly arranged.

**Result:**

As of January 2025, 101 patients have received at least one lecanemab infusion at West China Hospital (mean age: 66.32 ± 10.69; 30.69% male). Three patients received additional lecanemab treatment in Hainan prior to June 2024 via an early access program. Amyloid PET scans confirmed AD in all patients, 48 also underwent Tau PET (47.52%), and 50 had CSF testing (49.51%). Sixty‐one patients had MCI (60.40%) and forty had mild dementia (41.67%). Fifty‐four were ApoE ε4 heterozygotes (53.47%), and nine were ApoE ε4 homozygotes (8.91%). Forty‐three patients received intravenous prophylactic medications to prevent infusion reactions (42.57%), and seven patients experienced infusion reactions (6.93%). Ten patients (9.90%) had asymptomatic ARIA (8 with ARIA‐H, 2 with ARIA‐E). ARIA was observed at various infusion stages, with five cases in ApoE ε4 heterozygotes and five in noncarriers.

**Conclusion:**

Careful selection by MDT and amyloid PET confirmation ensured optimal recruitment. High‐field MRI and consistent radiologist interpretation facilitated ARIA detection. Our experience provides insights into establishing specialized medical frameworks for the safe and widespread administration of lecanemab.